# Supplementary figures and images for: TrxT and dhd are dispensable for Drosophila brain development but essential for l(3)mbt brain tumour growth
Source: EMBO Rep. 2024 May 15;25(7):6. doi: 10.1038/s44319-024-00154-1 (PMC11239866; doi:10.1038/s44319-024-00154-1)

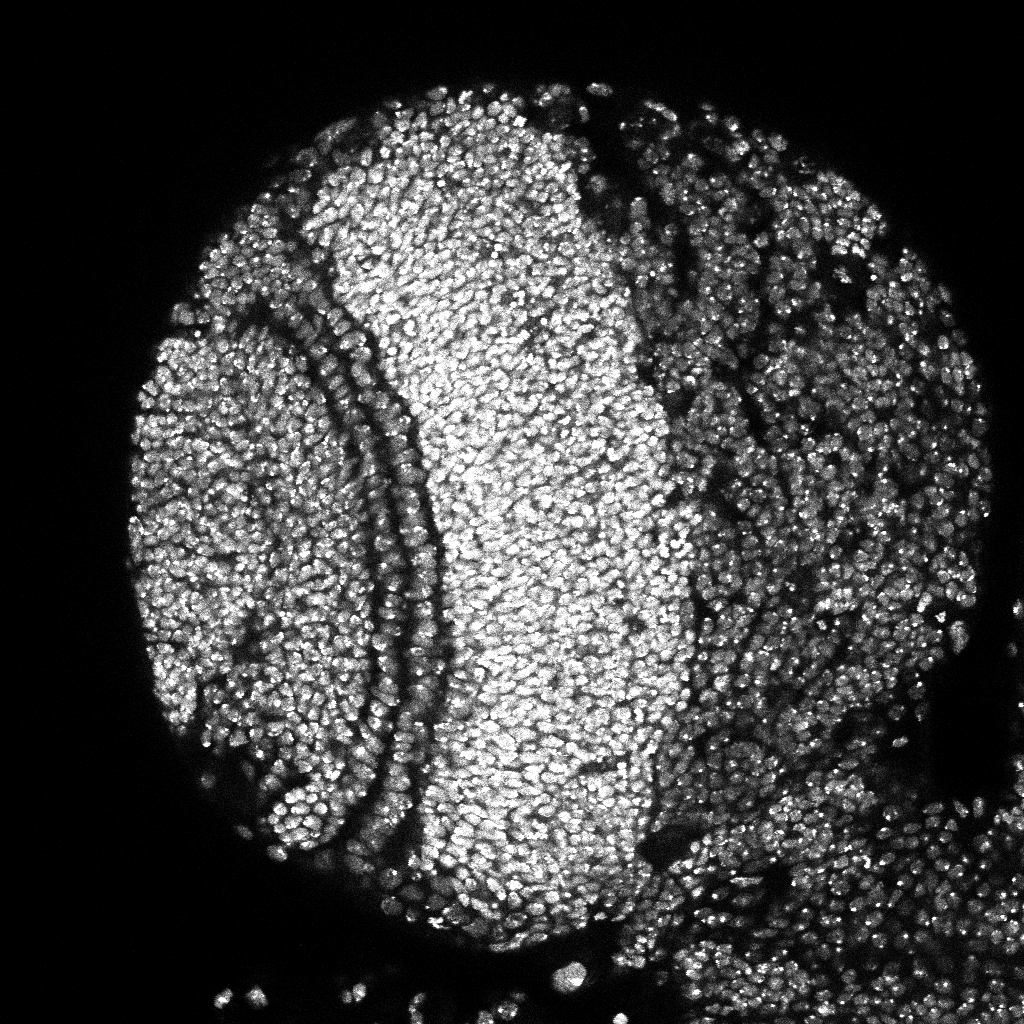

Supplement: Supplementary file 2 — Source data Fig. 1 [file 44319_2024_154_MOESM2_ESM.zip › 1C/DfJ5_DECadh_56d.tif]

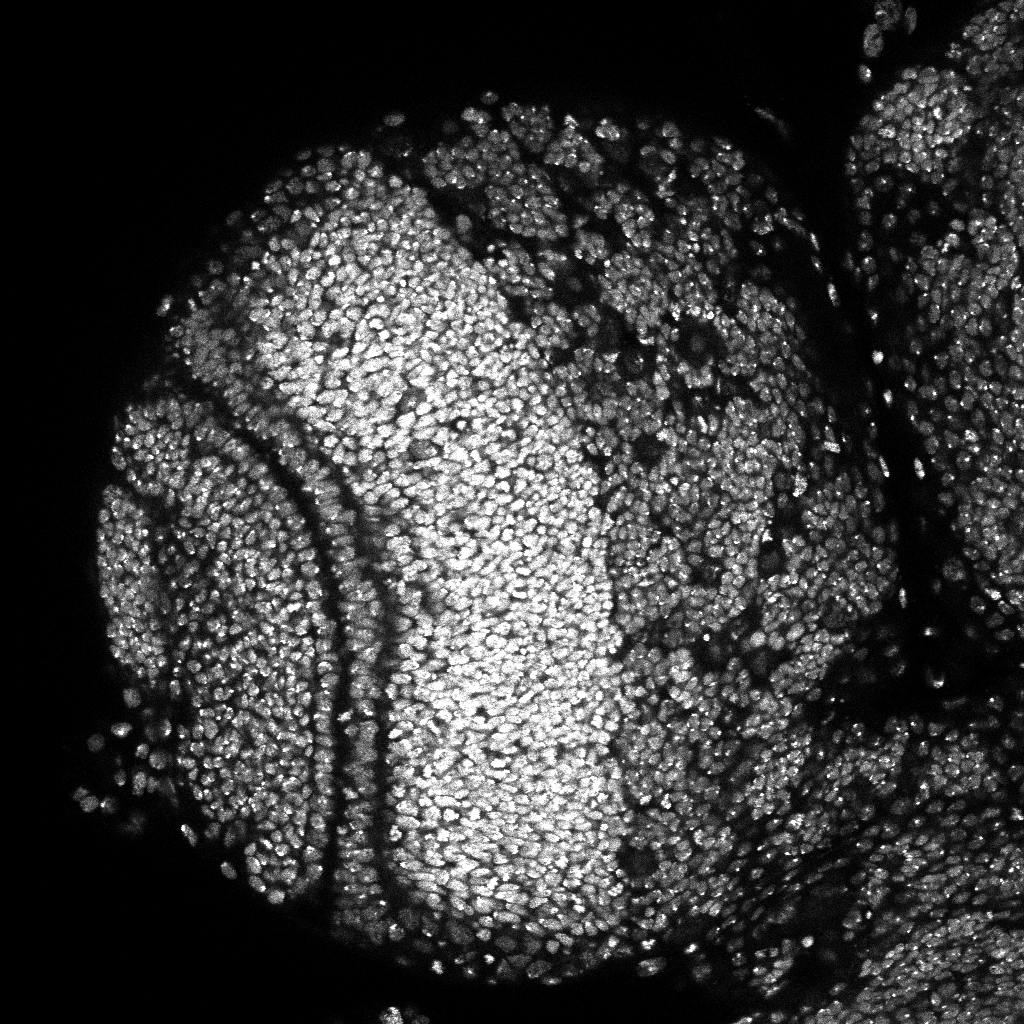

Supplement: Supplementary file 2 — Source data Fig. 1 [file 44319_2024_154_MOESM2_ESM.zip › 1C/dhdKO_DECadh_56d.tif]

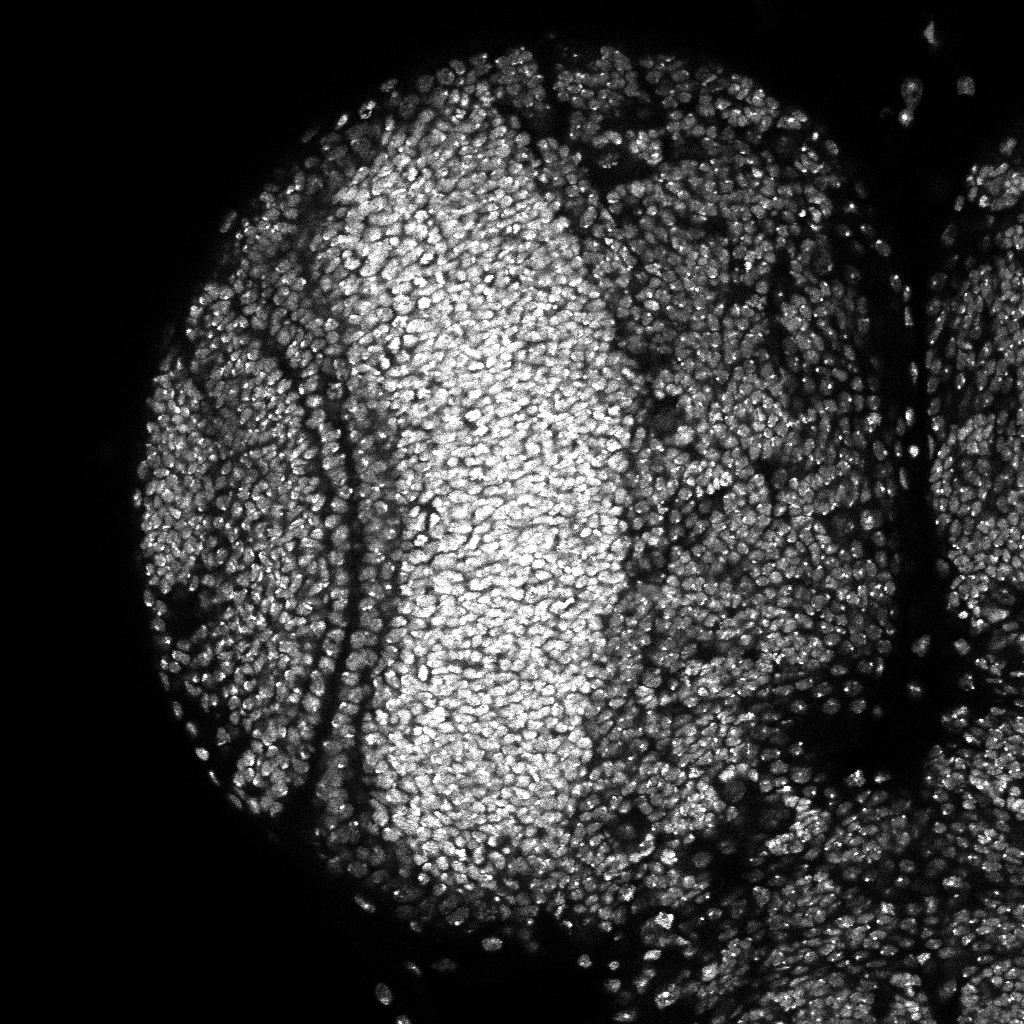

Supplement: Supplementary file 2 — Source data Fig. 1 [file 44319_2024_154_MOESM2_ESM.zip › 1C/TrxTKO_DECadh_56d.tif]

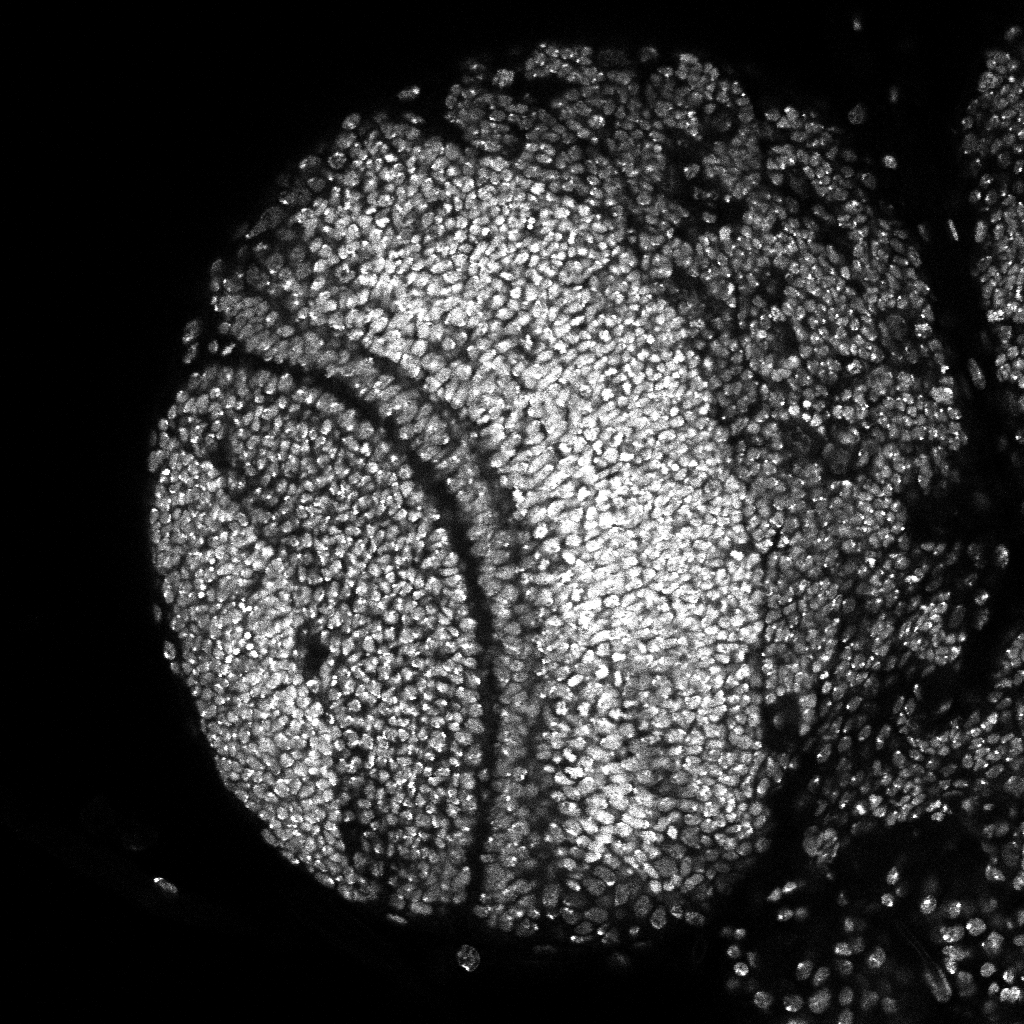

Supplement: Supplementary file 2 — Source data Fig. 1 [file 44319_2024_154_MOESM2_ESM.zip › 1C/w1118_DECadh_56d.tif]

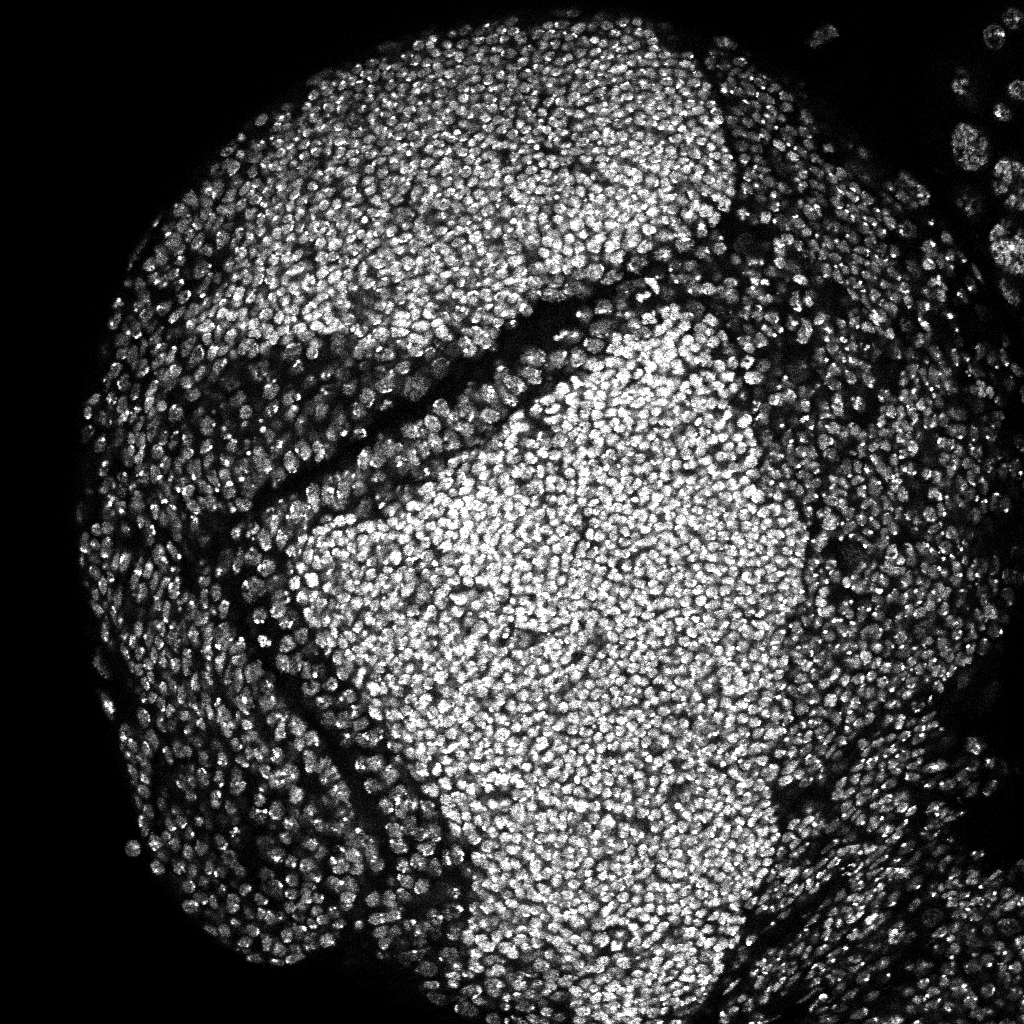

Supplement: Supplementary file 3 — Source data Fig. 2 [file 44319_2024_154_MOESM3_ESM.zip › 2B/male_DfJ5_mbt-ts1_DapiDECadh_1.tif]

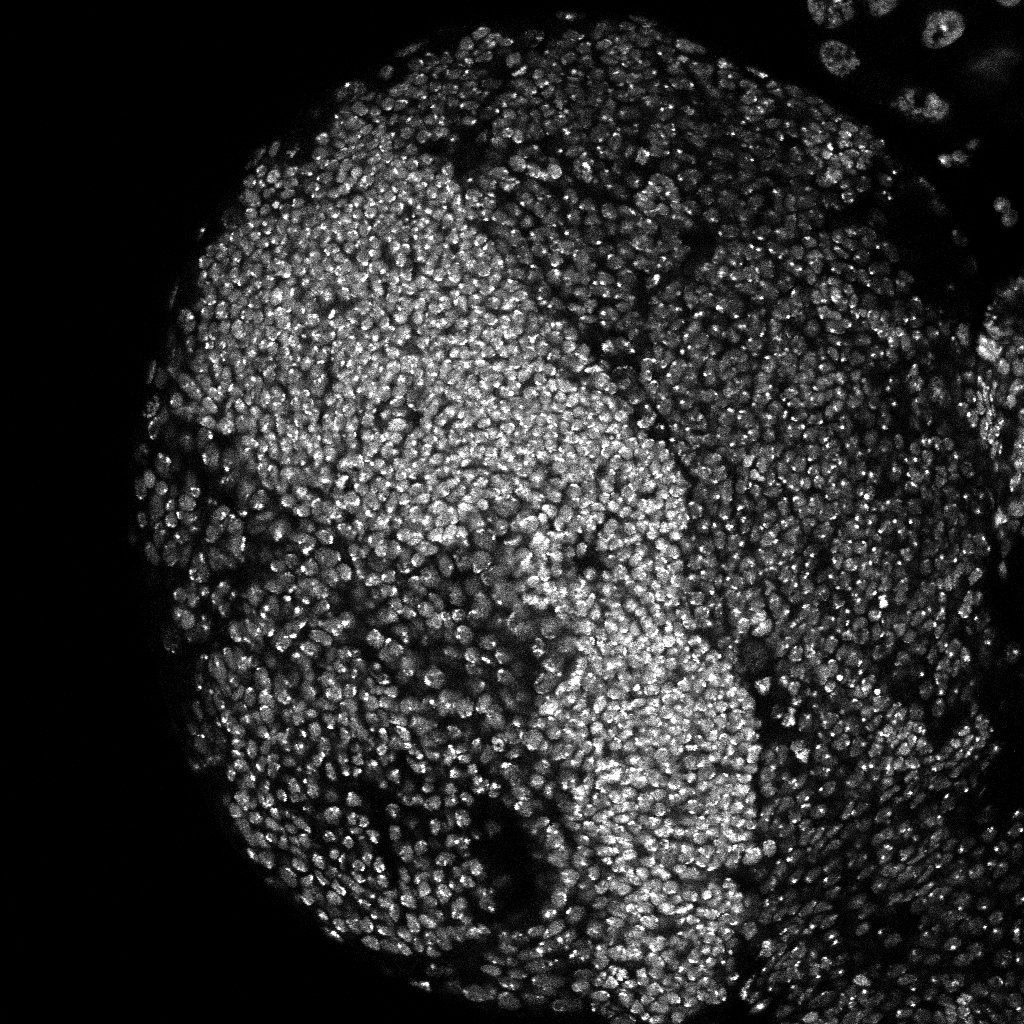

Supplement: Supplementary file 3 — Source data Fig. 2 [file 44319_2024_154_MOESM3_ESM.zip › 2B/male_DfJ5_mbt-ts1_DapiDECadh_2.tif]

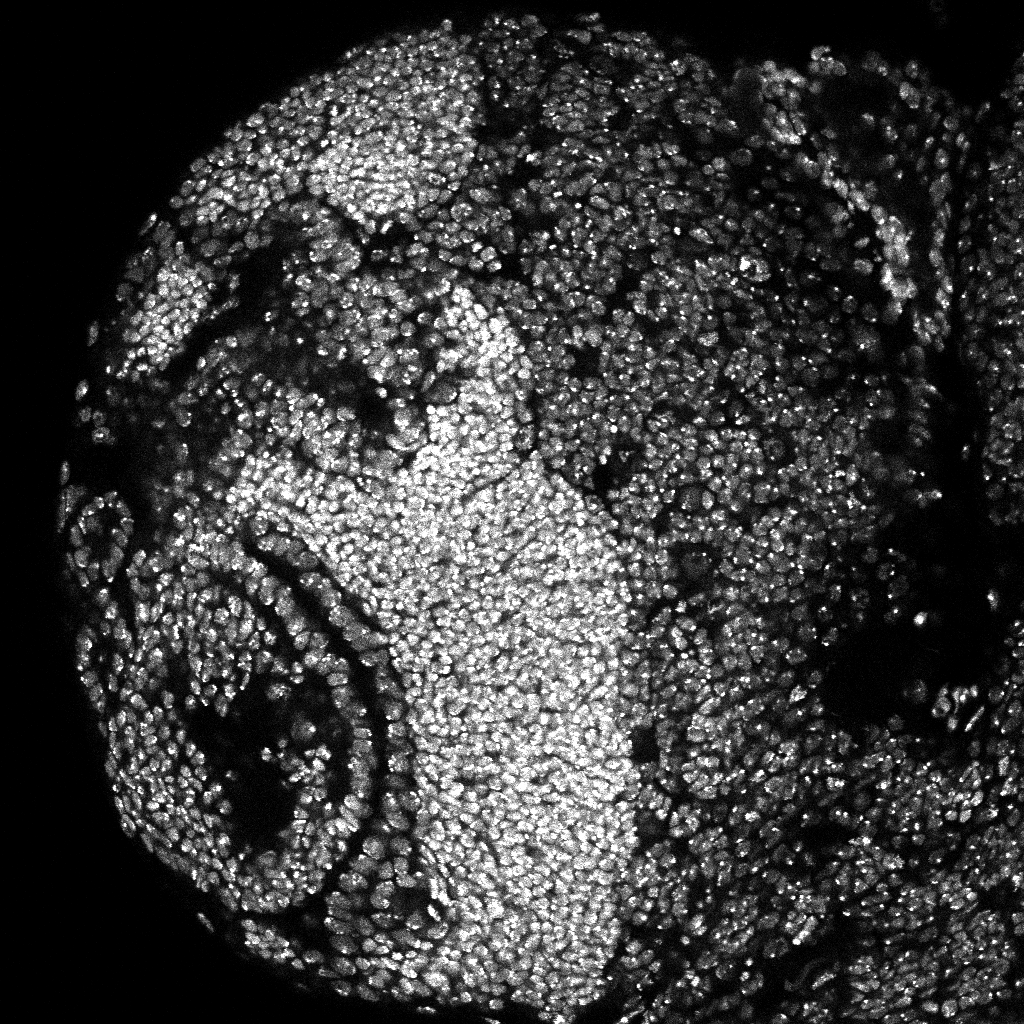

Supplement: Supplementary file 3 — Source data Fig. 2 [file 44319_2024_154_MOESM3_ESM.zip › 2B/male_DfJ5_mbt-ts1_DapiDECadh_3.tif]

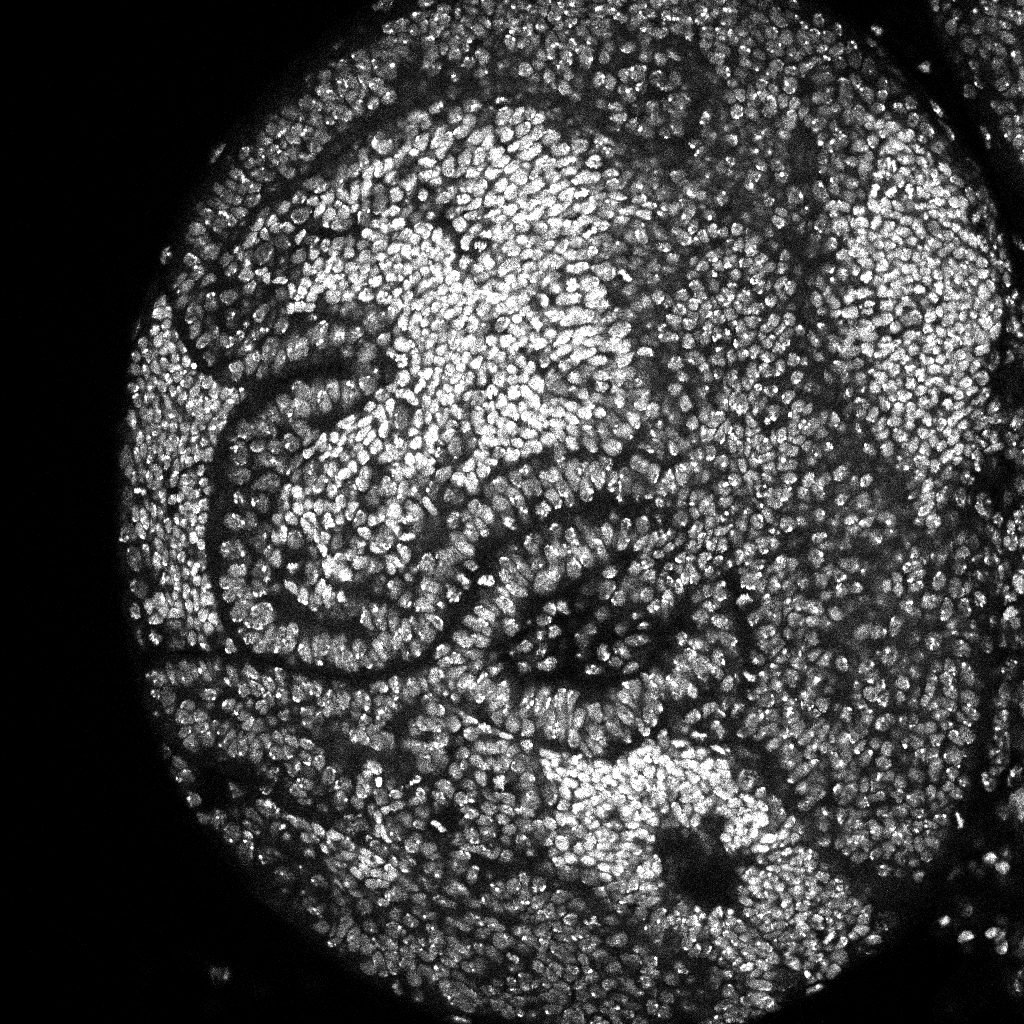

Supplement: Supplementary file 3 — Source data Fig. 2 [file 44319_2024_154_MOESM3_ESM.zip › 2B/male_mbt-ts1_DapiDECadh_1.tif]

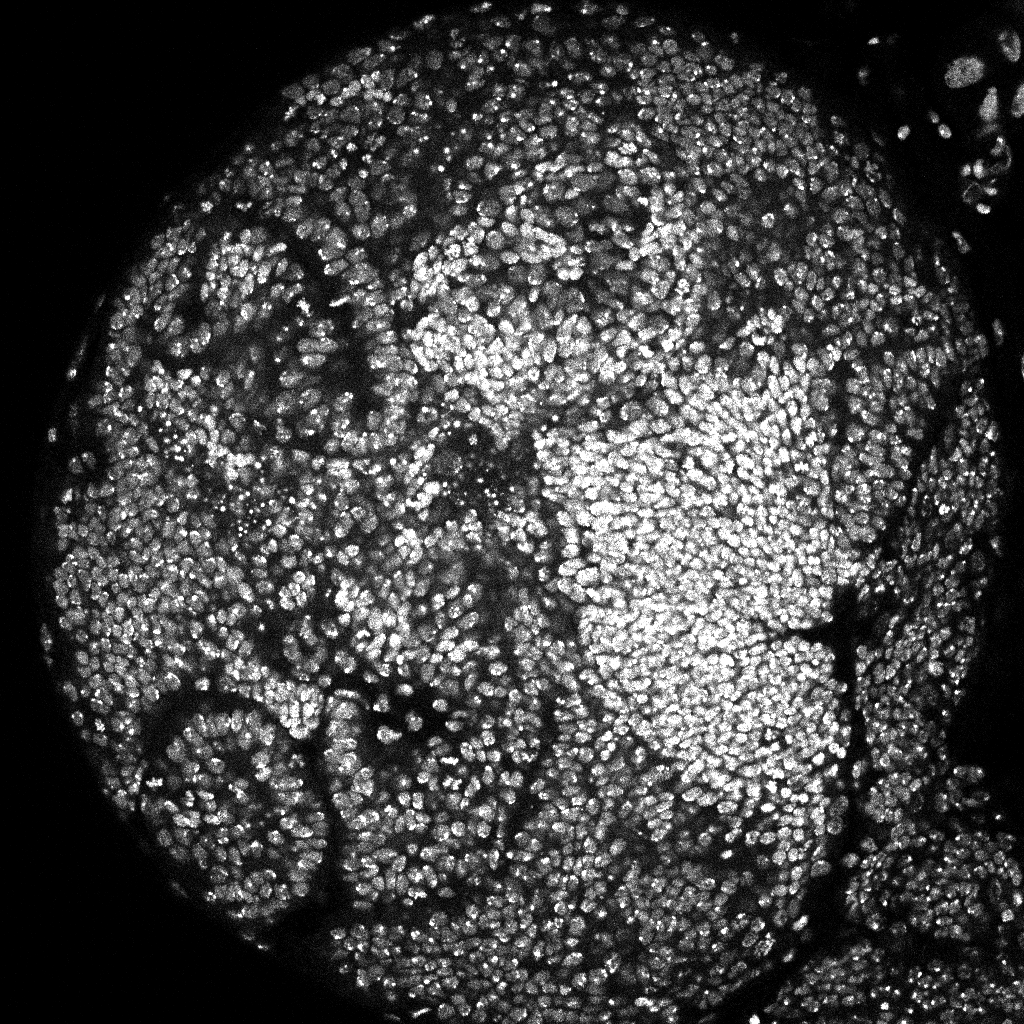

Supplement: Supplementary file 3 — Source data Fig. 2 [file 44319_2024_154_MOESM3_ESM.zip › 2B/male_mbt-ts1_DapiDECadh_2.tif]

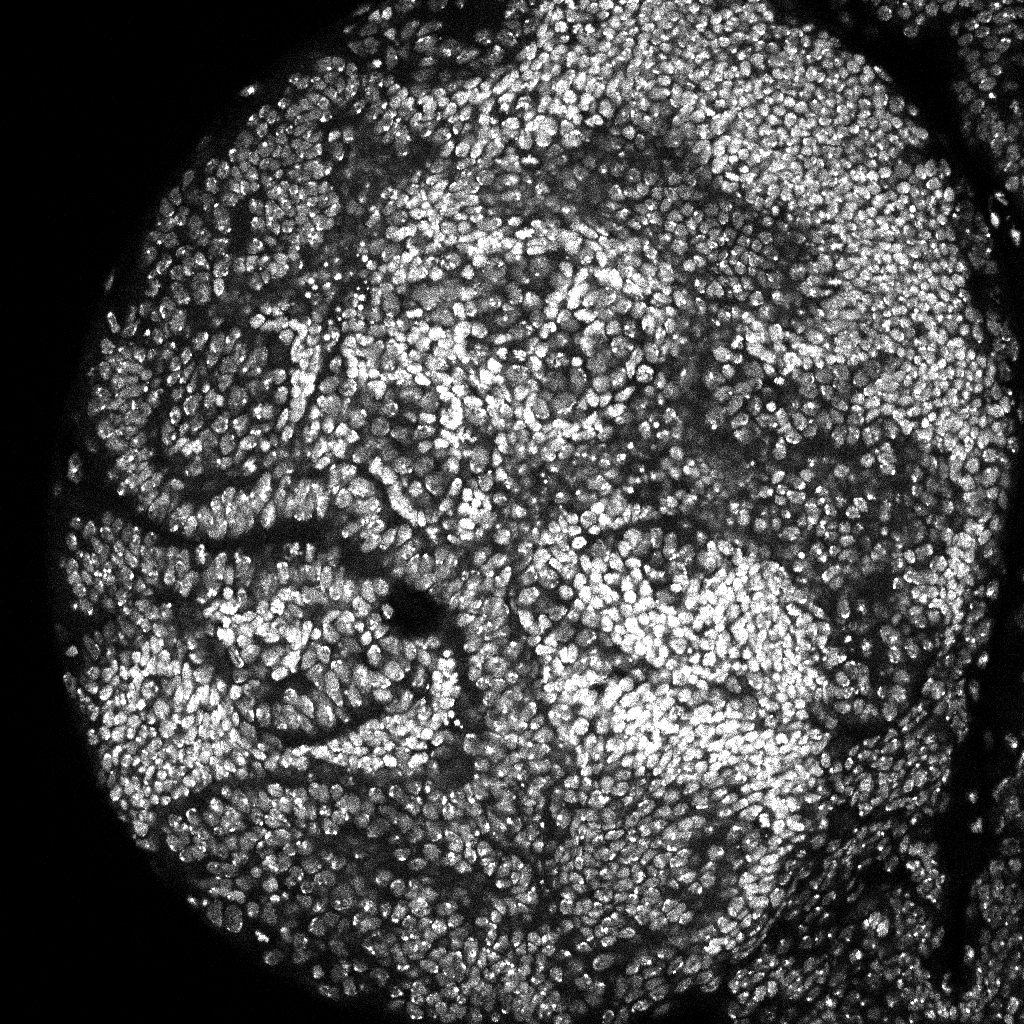

Supplement: Supplementary file 3 — Source data Fig. 2 [file 44319_2024_154_MOESM3_ESM.zip › 2B/male_mbt-ts1_DapiDECadh_3.tif]

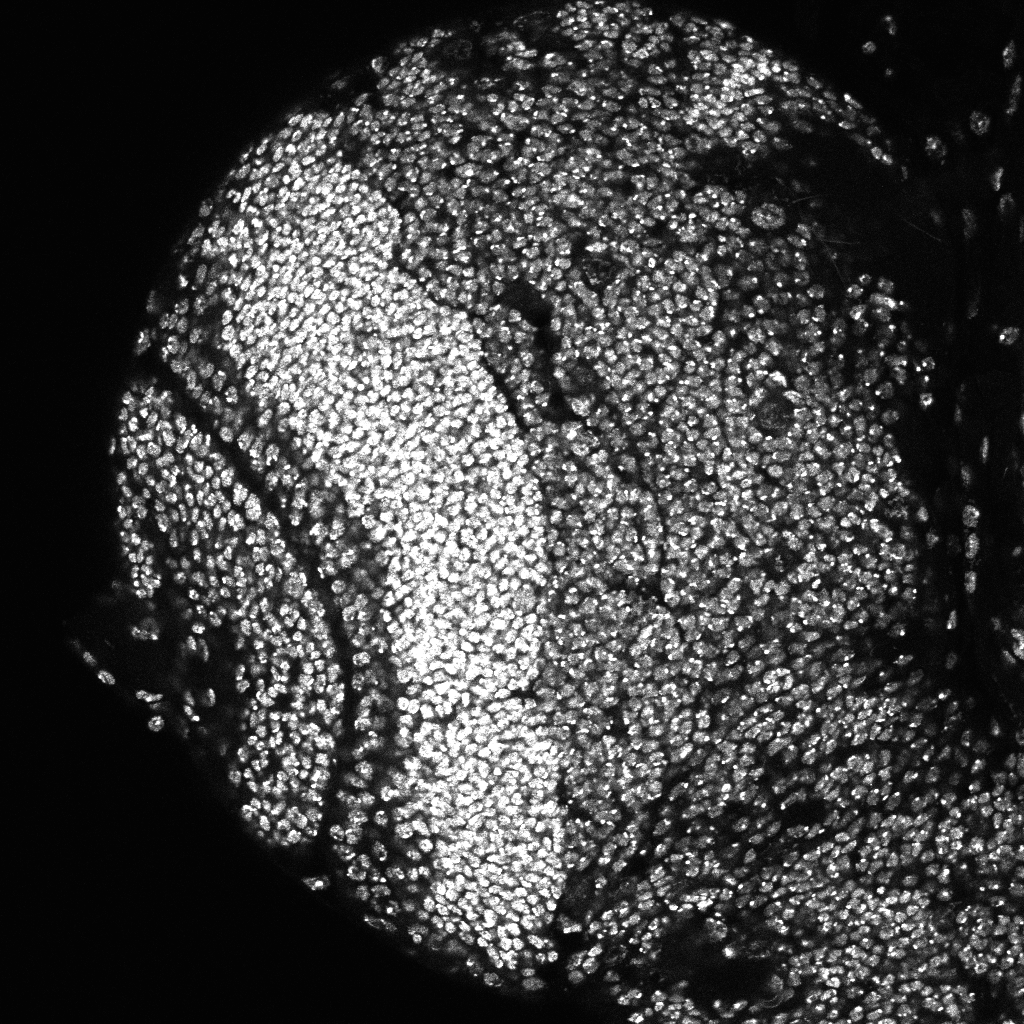

Supplement: Supplementary file 3 — Source data Fig. 2 [file 44319_2024_154_MOESM3_ESM.zip › 2B/male_w1118_DapiDECadh_2.tif]

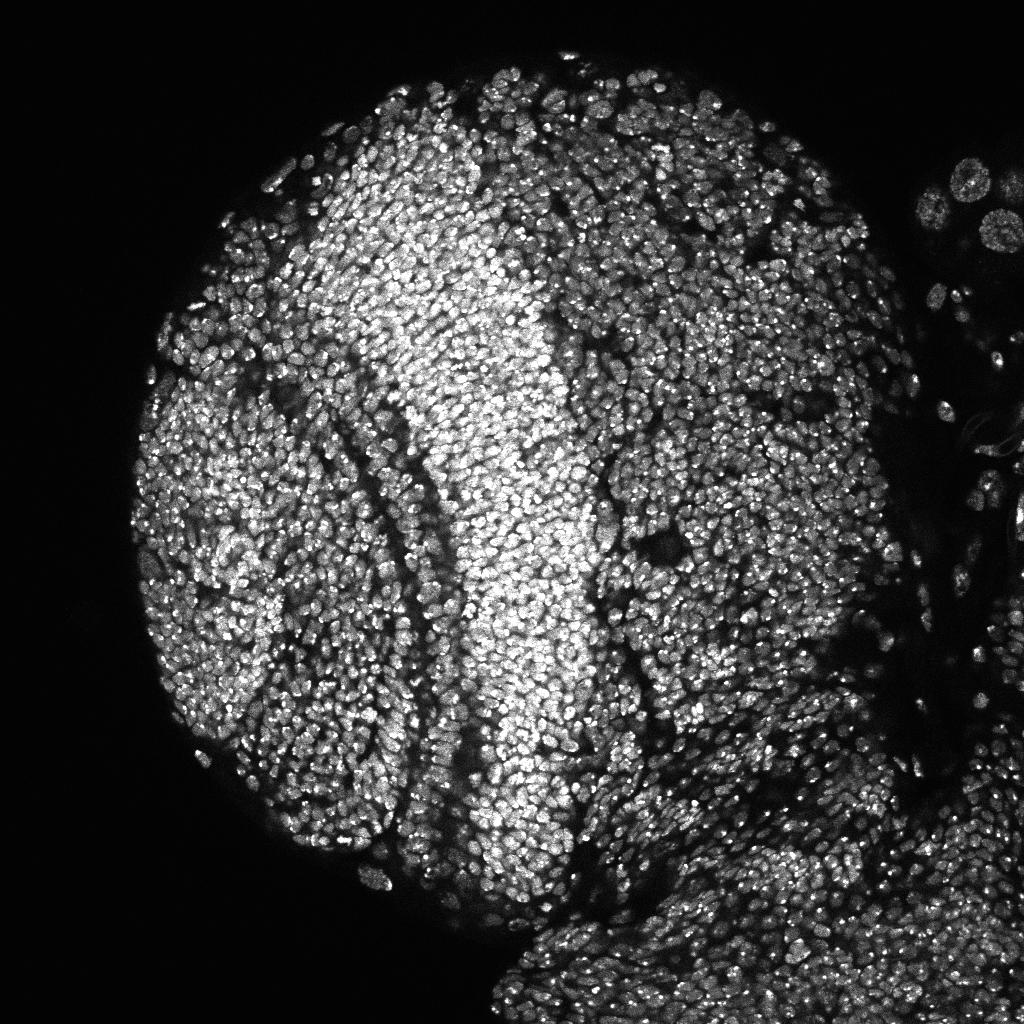

Supplement: Supplementary file 3 — Source data Fig. 2 [file 44319_2024_154_MOESM3_ESM.zip › 2B/male_w1118_DapiDECadh_3.tif]

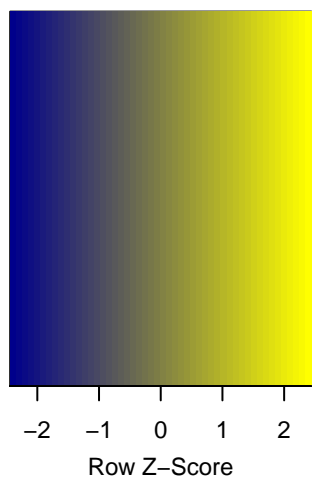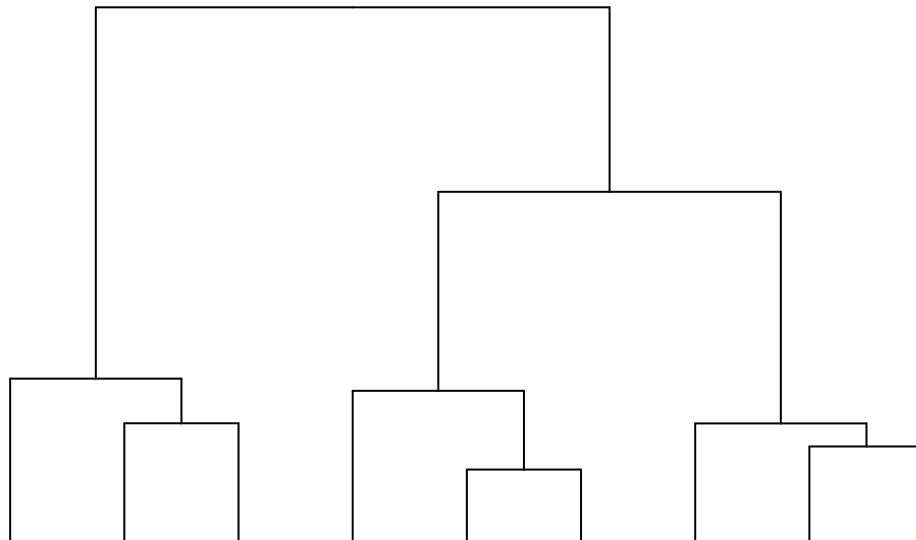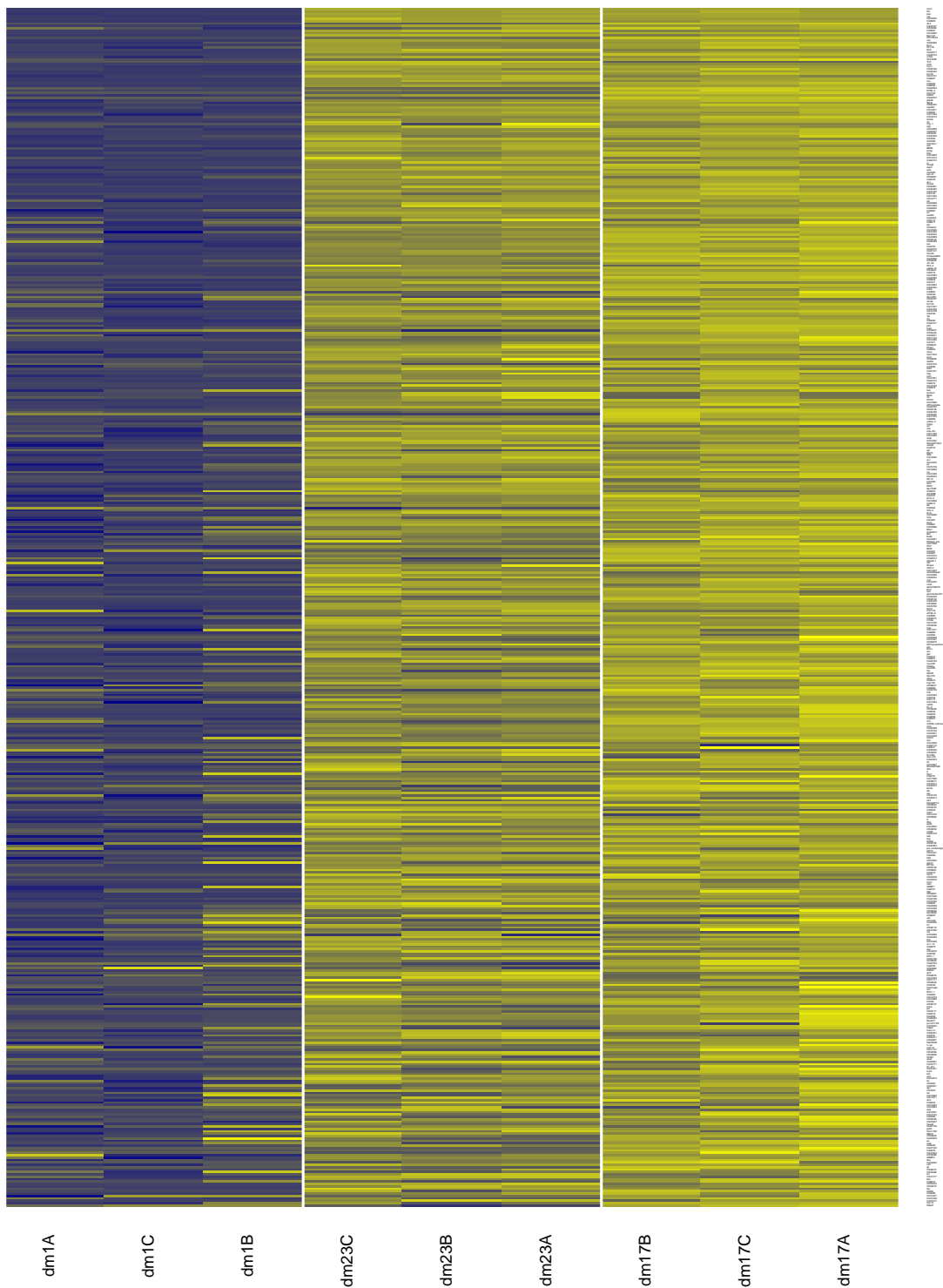

Supplement: Supplementary file 4 — Source data Fig. 3 [file 44319_2024_154_MOESM4_ESM.zip › 3D/Heatmaps/1_Rplot_males_dhd_byRank_dendroColumn.pdf]

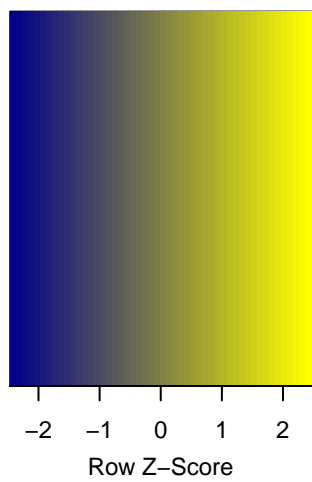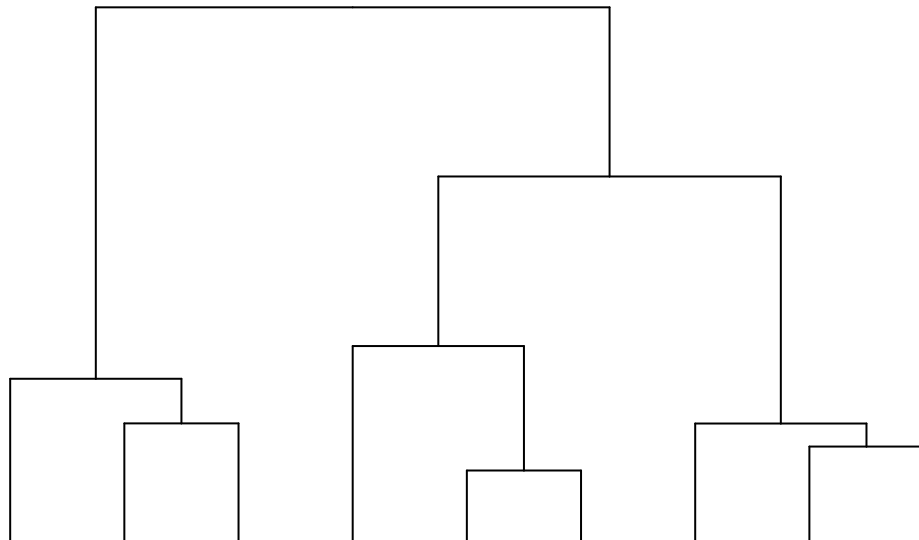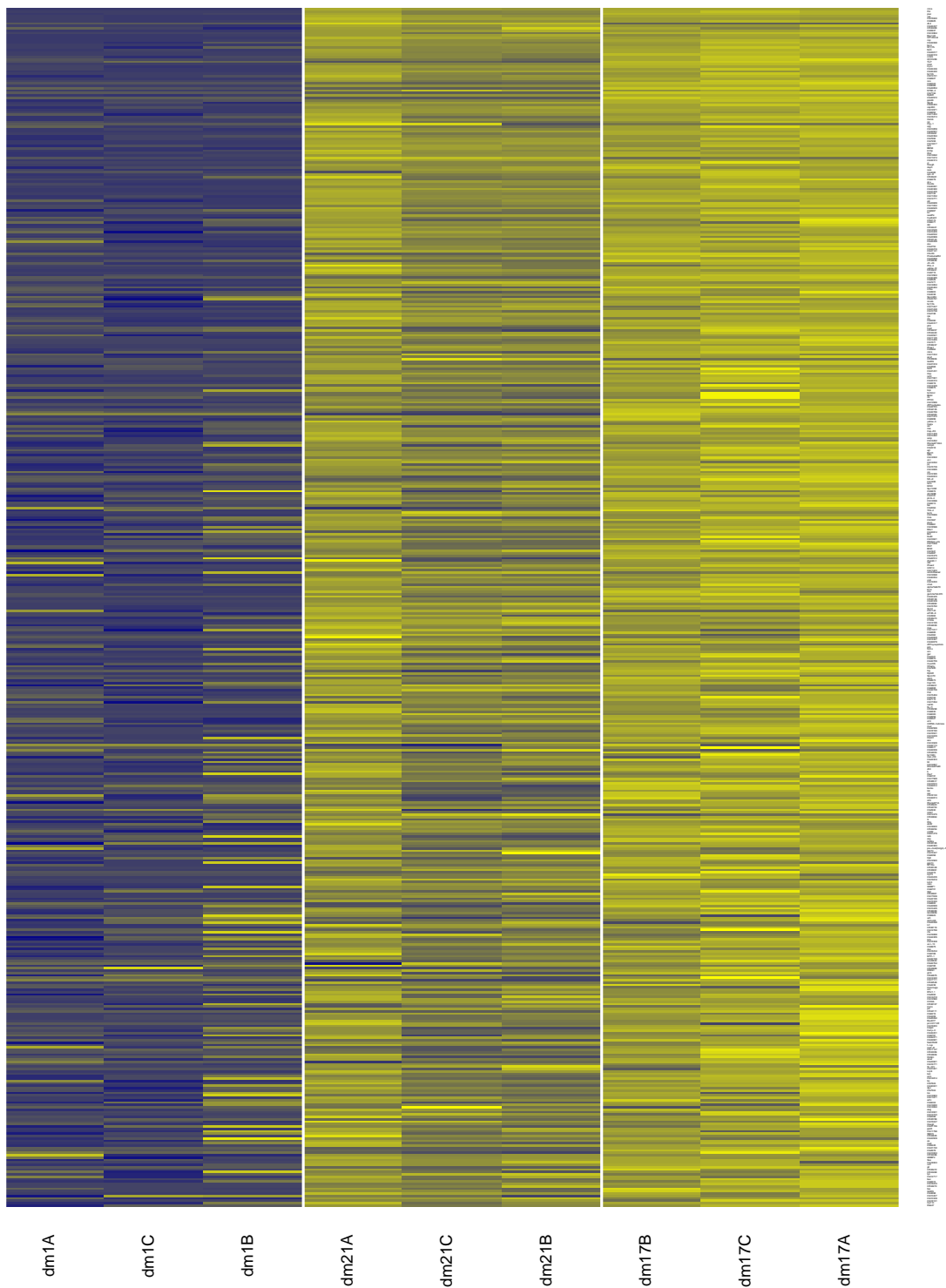

Supplement: Supplementary file 4 — Source data Fig. 3 [file 44319_2024_154_MOESM4_ESM.zip › 3D/Heatmaps/2_Rplot_males_TrxT_byRank_dendroColumn.pdf]

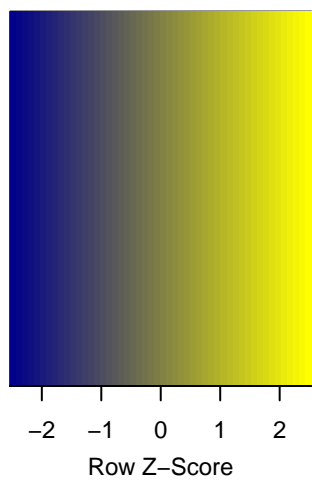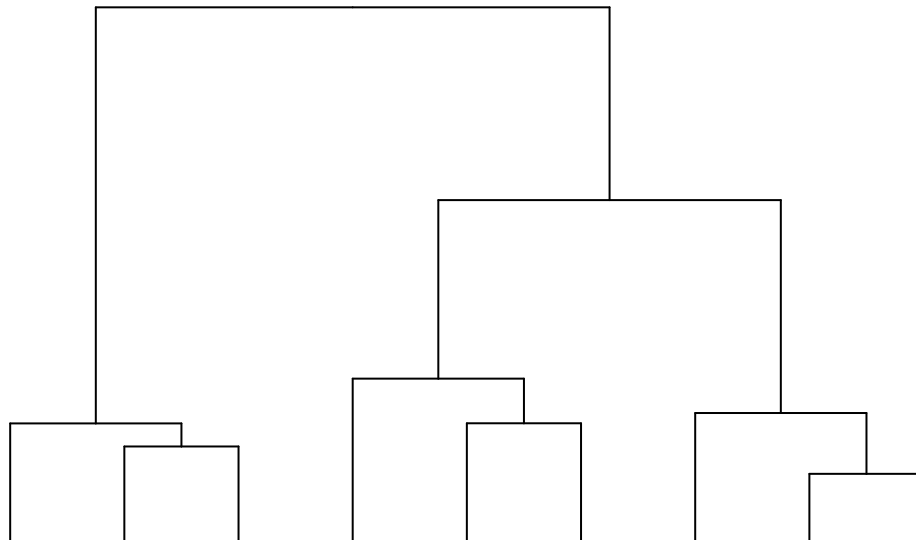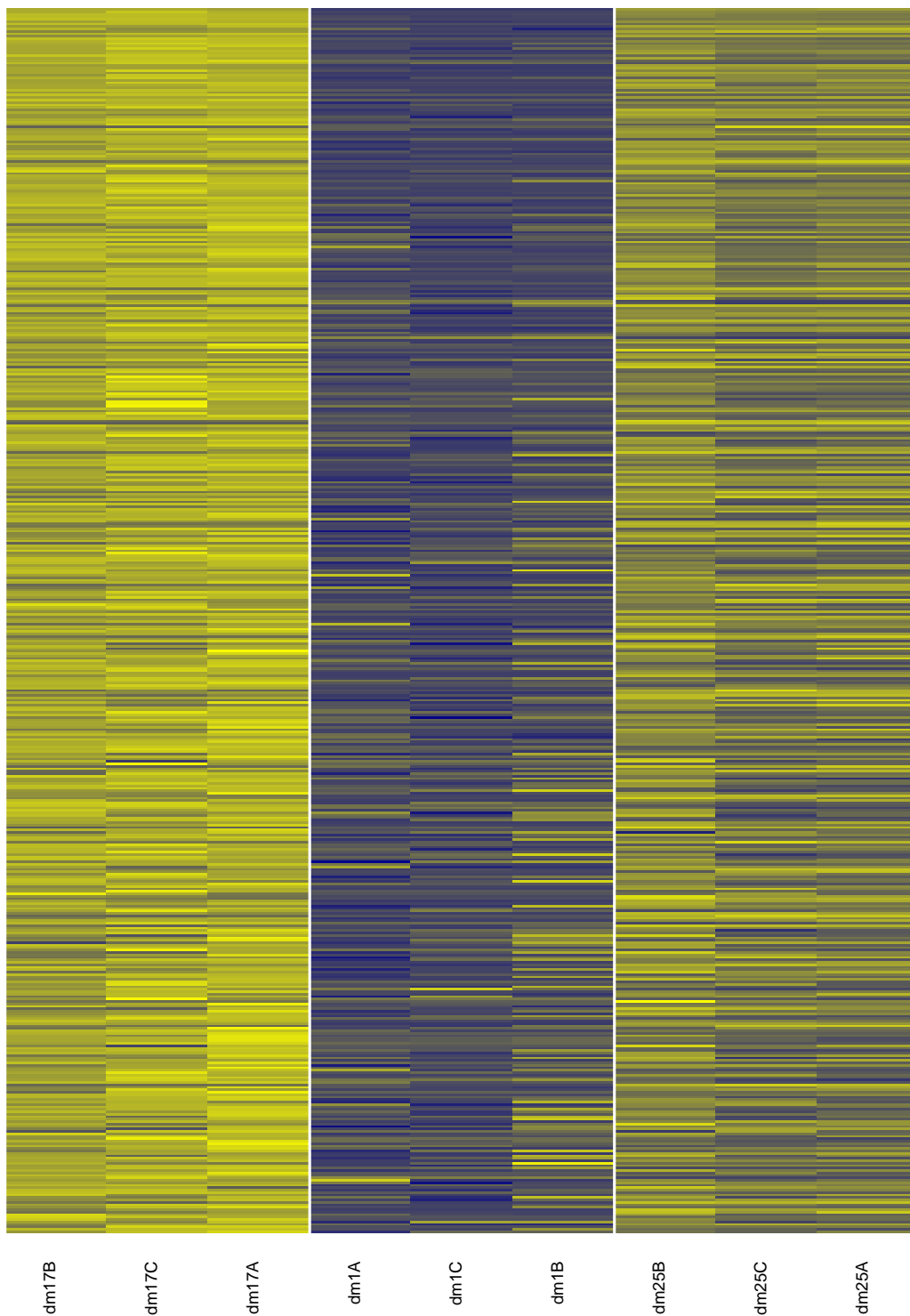

dm17B dm17C dm17A dm1A dm1C dm1B dm25B dm25C dm25A

Supplement: Supplementary file 4 — Source data Fig. 3 [file 44319_2024_154_MOESM4_ESM.zip › 3D/Heatmaps/3_Rplot_males_DfJ5_byRank_dendroColumn.pdf]
