# Supplementary material for: TrxT and dhd are dispensable for Drosophila brain development but essential for l(3)mbt brain tumour growth
Source: EMBO Rep. 2024 May 15;25(7):6. doi: 10.1038/s44319-024-00154-1 (PMC11239866; doi:10.1038/s44319-024-00154-1)
Supplement: Supplementary file 7 — Expanded View Figures [file 44319_2024_154_MOESM7_ESM.pdf]

Expanded View Figures

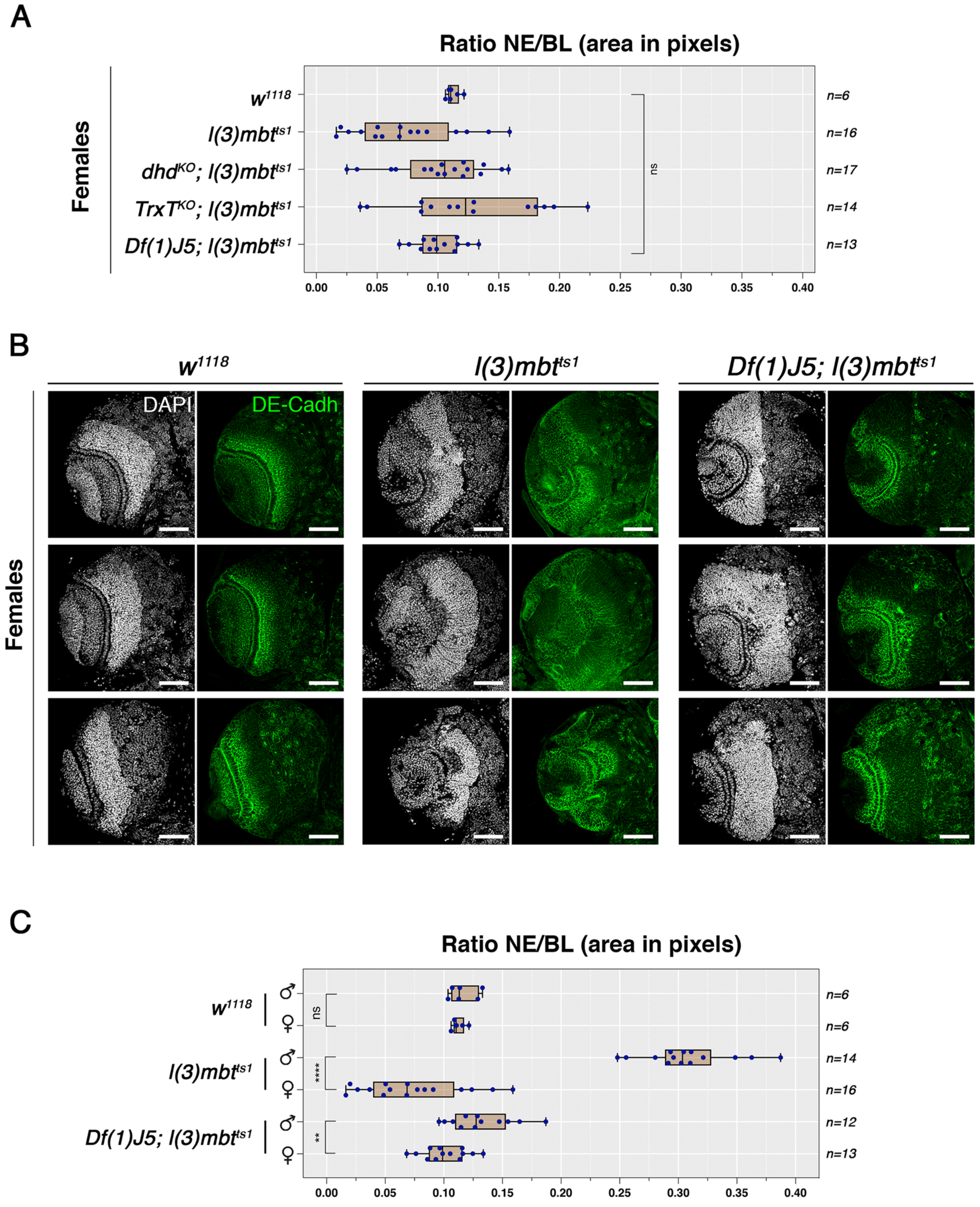

Figure EV1. ***TrxT* and *dhd* depletion affects female-specific mbt tumour traits.**

(**A**) Relative size of NE as a fraction of BL area in female control (*w*[1118]), *l(3)mbt*[ts1] single-mutant, and *dhd*[KO]*; l(3)mbt*[ts1], *TrxT*[KO]*; l(3)mbt*[ts1], *Df(1)J5; l(3)mbt*[ts1] double-mutant female larvae. Kruskal–Wallis test, ns $P > 0.05$. (**B**) Larval brain lobes from control (*w*[1118]), *l(3)mbt*[ts1] single-mutant, and *Df(1)J5; l(3)mbt*[ts1] double-mutant female larvae stained with DAPI (grey) and anti-DE-cadherin (green). Female *l(3)mbt*[ts1] lobes present shortened NE but retain a relatively normal CB and compact medulla (MED). Female *Df(1)J5; l(3)mbt*[ts1] lobes closely resemble wild-type brains. For each genotype, three representative different lobes are shown. Scale bar, 50 µm. (**C**) Relative size of NE as a fraction of brain lobe BL area in male and female control (*w*[1118]), *l(3)mbt*[ts1] single-mutant, and *Df(1)J5; l(3)mbt*[ts1] double-mutant larvae. Student's *t* test, ****$P < 0.0001$; **$P < 0.01$; ns $P > 0.05$. Data Information: (**A**, **C**) The vertical line within each box represents the median, and the box boundaries are defined by the 25th and 75th percentiles. The whiskers extend to the minimum and maximum values. Each blue dot represents a NE/BL ratio value (area in pixels) for a single brain lobe, with sample sizes indicated (**A**: $n = 6, 16, 17, 14, 13$; **C**: $n = 6, 6, 14, 16, 12, 13$). (**A**) Statistical analysis was performed using Kruskal–Wallis test, or (**C**) Student's *t* test. (**B**) Scale bar, 50 µm.

© The Author(s)

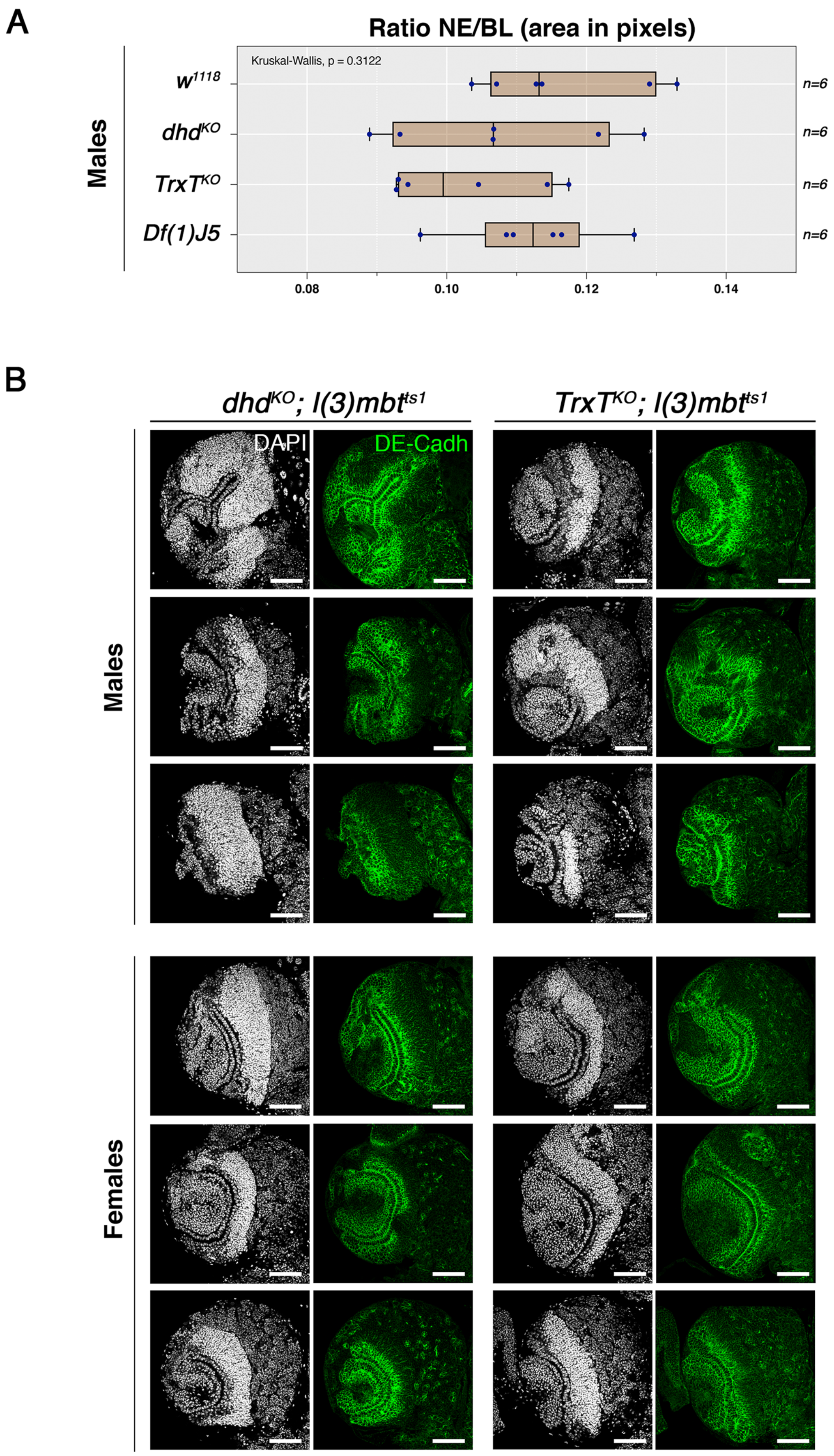
A
Ratio NE/BL (area in pixels)
Kruskal-Wallis, p = 0.3122
Males
w1118
dhdKO
TrxTKO
Df(1)J5
n=6
n=6
n=6
n=6
0.08
0.10
0.12
0.14
B
dhdKO; l(3)mbtts1
TrxTKO; l(3)mbtts1
DAPI
DE-Cadh
Males
Females

© The Author(s)

Figure EV2. *TrxT* and *dhd* depletion in wild-type and mbt tumour brains.

(**A**) Relative size of the NE as a fraction of BL area in male control (*w*^{1118}), *dhd*^{KO}, *TrxT*^{KO}, and *Df(1)J5* male larvae. Kruskal–Wallis test, ns $P > 0.05$. (**B**) Larval brain lobes from *dhd*^{KO}*; l(3)mbt*^{ts1} *and TrxT*^{KO}*; l(3)mbt*^{ts} male and female larvae stained with DAPI (grey) and anti-DE-cadherin (green). Lobes corresponding to the three best mbt-suppressed phenotype are shown for each genotype. Scale bar, 50 µm. Data Information: (**A**) The vertical line within each box represents the median, and the box boundaries are defined by the 25th and 75th percentiles. The whiskers extend to the minimum and maximum values. Each blue dot represents a NE/BL ratio value (area in pixels) for a single brain lobe, with sample sizes indicated ($n = 6$ in all samples). (**A**) Statistical analysis was performed using Kruskal–Wallis test. (**B**) Scale bar, 50 µm.

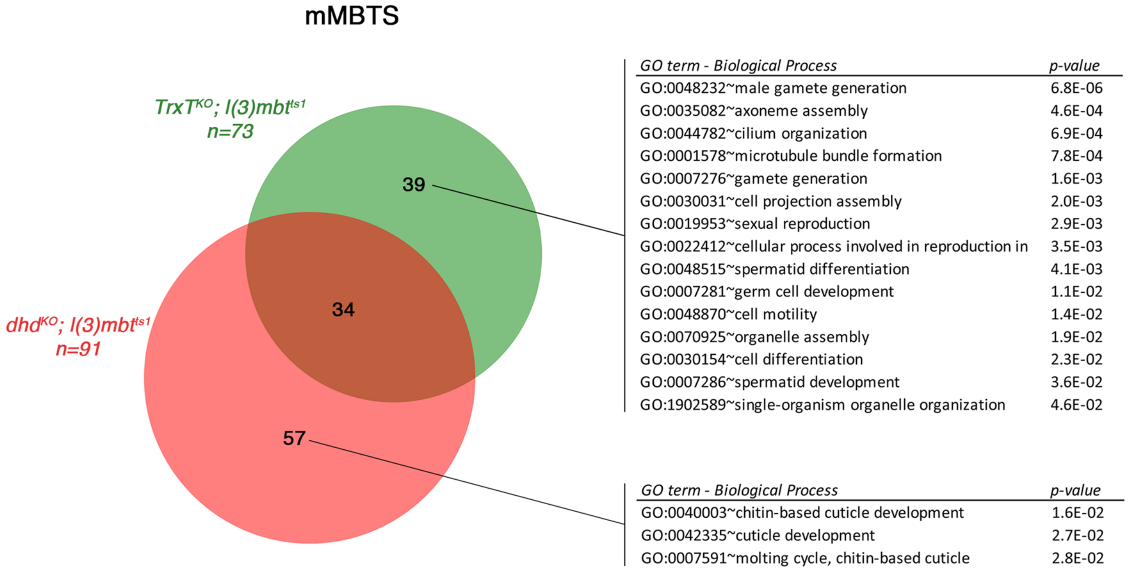

GO term - Biological Process	*p-value*
GO:0048232~male gamete generation	6.8E-06
GO:0035082~axoneme assembly	4.6E-04
GO:0044782~cilium organization	6.9E-04
GO:0001578~microtubule bundle formation	7.8E-04
GO:0007276~gamete generation	1.6E-03
GO:0030031~cell projection assembly	2.0E-03
GO:0019953~sexual reproduction	2.9E-03
GO:0022412~cellular process involved in reproduction in	3.5E-03
GO:0048515~spermatid differentiation	4.1E-03
GO:0007281~germ cell development	1.1E-02
GO:0048870~cell motility	1.4E-02
GO:0070925~organelle assembly	1.9E-02
GO:0030154~cell differentiation	2.3E-02
GO:0007286~spermatid development	3.6E-02
GO:1902589~single-organism organelle organization	4.6E-02

GO term - Biological Process	*p-value*
GO:0040003~chitin-based cuticle development	1.6E-02
GO:0042335~cuticle development	2.7E-02
GO:0007591~molting cycle, chitin-based cuticle	2.8E-02

Figure EV3. mMBTS genes affected by depletion of *TrxT* or *dhd* in mbt tumours.

Venn diagram showing the number of genes of the mMBTS downregulated in males dhd^{KO}; $l(3)mbt^{ts1}$ (red) and $TrxT^{KO}$; $l(3)mbt^{ts1}$(green) compared to $l(3)mbt^{ts1}$. Gene Ontology terms that are significantly enriched in the group of genes downregulated in $TrxT^{KO}$; $l(3)mbt^{ts1}$ only, and in dhd^{KO}; $l(3)mbt^{ts1}$ only ($P < 0.05$). No significantly enriched GOs were identified in the overlapping genes. Data Information: Venn Diagram was performed using BioVenn online tool, and Gene Ontology terms were analysed using DAVID 2021 online tool. Statistical analysis performed by DAVID was Fisher's Exact test.

© The Author(s)

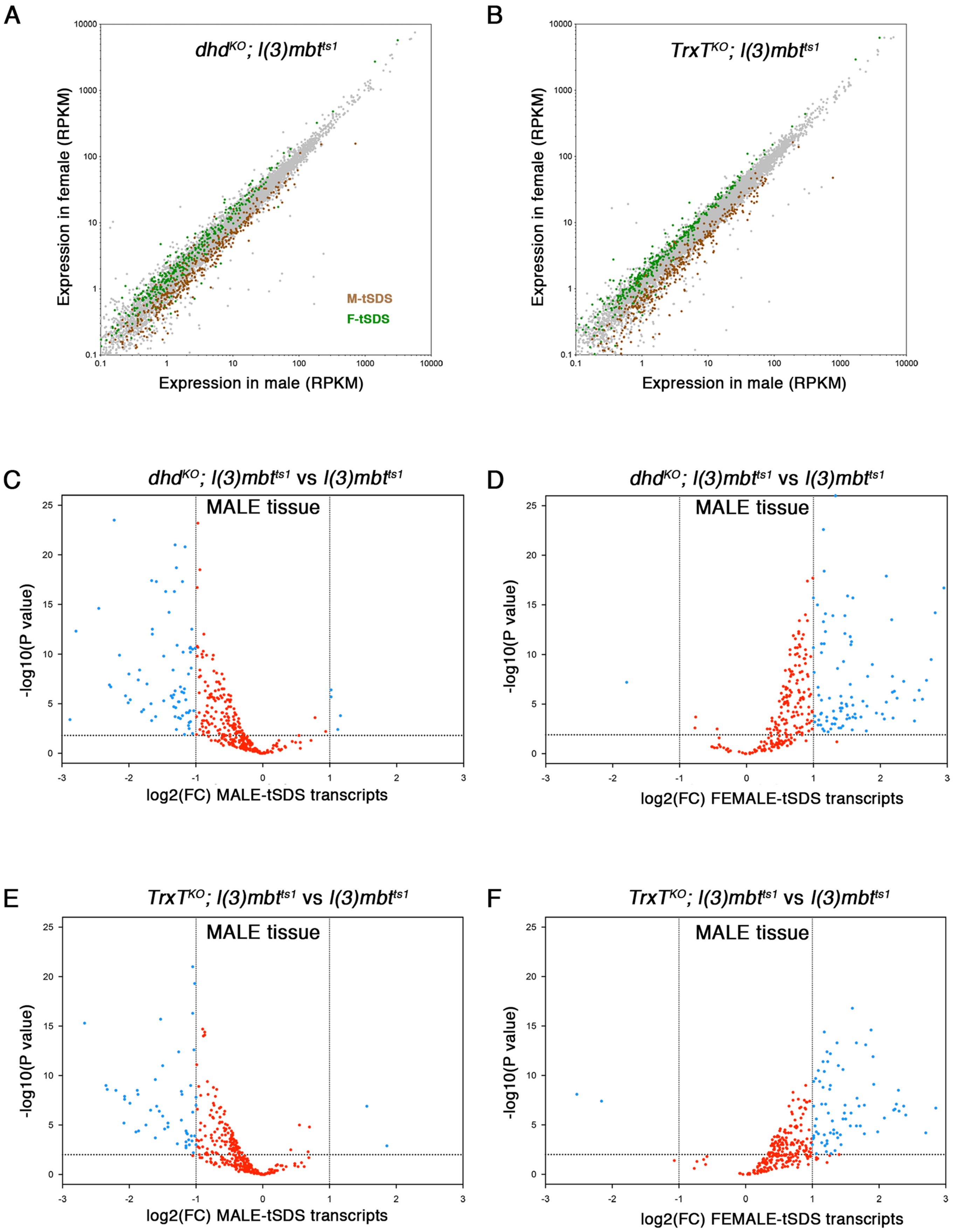
A
dhdKO; l(3)mbtts1
Expression in female (RPKM)
Expression in male (RPKM)
M-tSDS
F-tSDS
B
TrxTKO; l(3)mbtts1
Expression in female (RPKM)
Expression in male (RPKM)
C
dhdKO; l(3)mbtts1 vs l(3)mbtts1
MALE tissue
-log10(P value)
log2(FC) MALE-tSDS transcripts
D
dhdKO; l(3)mbtts1 vs l(3)mbtts1
MALE tissue
-log10(P value)
log2(FC) FEMALE-tSDS transcripts
E
TrxTKO; l(3)mbtts1 vs l(3)mbtts1
MALE tissue
-log10(P value)
log2(FC) MALE-tSDS transcripts
F
TrxTKO; l(3)mbtts1 vs l(3)mbtts1
MALE tissue
-log10(P value)
log2(FC) FEMALE-tSDS transcripts

© The Author(s)

Figure EV4. Expression of mbt SDS genes in *dhd*^{KO}; *l(3)mbt*^{ts1} and *TrxT*^{KO}; *l(3)mbt*^{ts1} double-mutant larvae.

(**A**, **B**) Plots showing the expression level of transcripts in male (*x* axis) and female (*y* axis) samples from *dhd*^{KO}; *l(3)mbt*^{ts1} (**A**) and *TrxT*^{KO}; *l(3)mbt*^{ts1} (**B**) larvae. Green and brown dots correspond to genes that are significantly overexpressed in male versus female (M-tSDS) and female versus male (F-tSDS) mbt tumours, respectively. Grey dots correspond to genes that are expressed at levels that are not significantly different between males and females. (**C–F**) Volcano plots showing the significance of the fold change in expression levels of the M-tSDS (**C**, **E**) and F-tSDS (**D**, **F**) genes between *dhd*^{KO}; *l(3)mbt*^{ts1} and *l(3)mbt*^{ts1} (**C**, **D**) and *TrxT*^{KO}; *l(3)mbt*^{ts1} and *l(3)mbt*^{ts1} (**E**, **F**) male samples. Blue dots represent genes that are significantly (FDR = 0.05) upregulated (log2(FC) > 1) or downregulated (log2(FC < -1). Genes whose differential expression is not significant are coloured in red. Data Information: (**A**, **B**) Expression levels of transcripts correspond to RPKM values. (**C–F**) Differential expression analysis was performed using *edgeR*, which applies the generalised linear model (GLM) likelihood ratio statistical test. Genes with absolute FC > 2 and FDR < 0.05 were considered differentially expressed.

 © The Author(s)

A

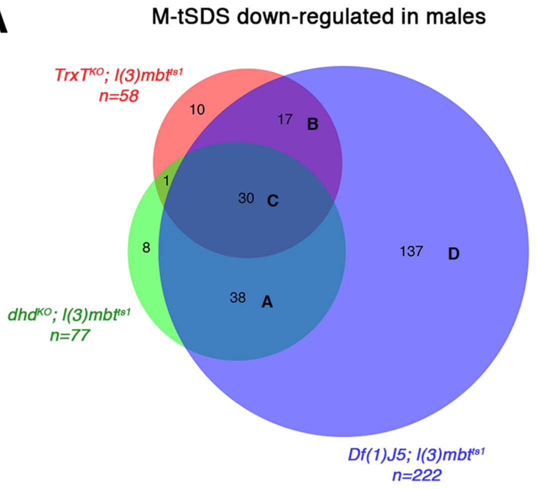

GO term - Biological Processes	*p-value*
**GROUP D (137 genes)**	
GO:0050767~regulation of neurogenesis	2.31E-05
GO:0090251~protein localization involved in establishment of planar polarity	3.60E-05
GO:0042067~establishment of ommatidial planar polarity	8.08E-05
GO:0007156~homophilic cell adhesion via plasma membrane adhesion molecules	1.27E-04
GO:0007400~neuroblast fate determination	1.55E-04
GO:0061331~epithelial cell proliferation involved in Malpighian tubule morphogenesis	1.65E-04
GO:0007422~peripheral nervous system development	1.86E-04
GO:0007417~central nervous system development	2.20E-04
GO:0007423~sensory organ development	2.88E-04
GO:0043065~positive regulation of apoptotic process	7.29E-04
GO:0045944~positive regulation of transcription from RNA polymerase II promoter	8.53E-04
GO:0007419~ventral cord development	9.65E-04
GO:0016339~calcium-dependent cell-cell adhesion via plasma membrane cell adhesion molecules	1.61E-03
GO:0001736~establishment of planar polarity	1.61E-03
GO:0007155~cell adhesion	1.62E-03
GO:0042051~compound eye photoreceptor development	2.32E-03
GO:0007476~imaginal disc-derived wing morphogenesis	2.76E-03
GO:0035159~regulation of tube length, open tracheal system	3.18E-03
GO:0061382~Malpighian tubule tip cell differentiation	3.60E-03
GO:0046667~compound eye retinal cell programmed cell death	3.60E-03
GO:0016318~ommatidial rotation	3.86E-03
GO:0001708~cell fate specification	3.86E-03
GO:0010888~negative regulation of lipid storage	4.47E-03
GO:2000177~regulation of neural precursor cell proliferation	4.47E-03
GO:0030050~vesicle transport along actin filament	4.47E-03
GO:0030182~neuron differentiation	4.94E-03
GO:0035332~positive regulation of hippo signaling	7.39E-03
GO:0045746~negative regulation of Notch signaling pathway	8.50E-03
GO:0044331~cell-cell adhesion mediated by cadherin	8.80E-03
GO:0006357~regulation of transcription from RNA polymerase II promoter	9.95E-03

B

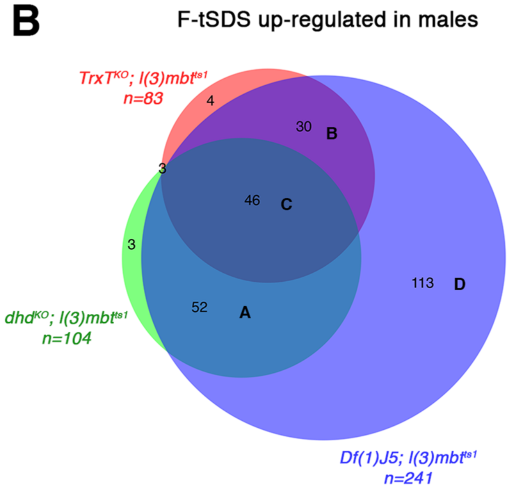

GO term - Biological Processes	*p-value*
**GROUP B (30 genes)**	
GO:0046839~phospholipid dephosphorylation	7.23E-07
GO:0006644~phospholipid metabolic process	1.94E-05
GO:0007602~phototransduction	4.88E-03
**GROUP D (113 genes)**	
GO:0050808~synapse organization	6.76E-05
GO:0045433~male courtship behavior, veined wing generated song producti	1.45E-04
GO:0007186~G-protein coupled receptor signaling pathway	8.31E-04
GO:0002121~inter-male aggressive behavior	6.26E-03
GO:0007218~neuropeptide signaling pathway	9.94E-03

Figure EV5. mbt SDS genes affected by depletion of *TrxT* and *dhd* in mbt tumours.

Venn diagrams and Gene Ontology terms that are significantly enriched ($P < 0.01$) in the downregulated M-tSDS (**A**) and upregulated F-tSDS (**B**) genes in *dhd*^{KO}; *l(3)mbt*^{ts1} (green) and *TrxT*^{KO}; *l(3)mbt*^{ts1}(red) and *Df(1)J5; l(3)mbt*^{ts1} (blue) male larvae. A = overlapping genes between *dhd*^{KO}; *l(3)mbt*^{ts1} and *Df(1)J5; l(3)mbt*^{ts1}; B = overlapping genes between *TrxT*^{KO}; *l(3)mbt*^{ts1} and *Df(1)J5; l(3)mbt*^{ts1}; C = overlapping genes between *dhd*^{KO}; *l(3)mbt*^{ts1}, *TrxT*^{KO}; *l(3)mbt*^{ts1}, and *Df(1)J5; l(3)mbt*^{ts1}; D = genes specifically affected in *Df(1)J5; l(3)mbt*^{ts1}. Data Information: Venn Diagrams were performed using BioVenn online tool, and Gene Ontology terms were analysed using DAVID 2021 online tool. Statistical analysis performed by DAVID was Fisher's Exact test.
